# Hypoxia-induced nuclear localization of ubiquinol-cytochrome-c reductase complex assembly factor 3 (UQCC3) in hepatocellular carcinoma

**DOI:** 10.1038/s41392-024-01909-x

**Published:** 2024-07-29

**Authors:** Yun Yang, Yinhao Wei, Yiying Sun, Yong Zhou, Hanshuo Yang

**Affiliations:** 1 Department of Biotherapy, Cancer Center and State Key Laboratory of Biotherapy, West China Hospital, Sichuan University, Chengdu, 610041 China; 2 Department of Gastrointestinal Surgery, West China Hospital, Sichuan University, Chengdu, 610041 China

**Keywords:** Cancer microenvironment, Prognostic markers

**Dear Editor,**

The mitochondrion plays crucial roles in cellular energy generation and signaling transduction. Increasing studies have shown that nuclear-encoded mitochondrial proteins translocate into the nucleus to regulate cell function and behavior, known as retrograde pathway, through which mitochondria communicate their status to the nucleus.^1^ Mitochondrial-nuclear communication represents a mechanism contributing to cellular adaptation to various stresses.^2^ However, the underlying mechanisms of mitochondrial retrograde signaling remain not fully understood.

Our previous studies showed that UQCC3 plays an indispensable role in promoting the adaptation of hepatocellular carcinoma (HCC) cells to hypoxia. Increased UQCC3 expression affects bioenergetic reprogramming of HCC cells under hypoxia by simultaneously regulating oxidative phosphorylation and glycolysis.^3^ In this study, we revealed that UQCC3 was translocated into the nucleus under hypoxic stress, was pivotal for HCC cells adapting to hypoxia, and contributed to adverse clinical outcomes in patients with HCC.

We previously reported that hypoxic stress increases UQCC3 expression in HCC cells.^3^ Here, we detected the location of UQCC3 in live HCC cells under hypoxic stress. Immunofluorescent imaging showed that UQCC3-EGFP fusion protein was predominantly localized in mitochondria without nuclear presence under normoxia. Under hypoxia, it was distinctly detected in the nucleus and mitochondria. Z-stacked imaging and quantitative fluorescence analysis also revealed the nuclear localization of UQCC3 protein in hypoxic HCC cells (Fig. 1a). Fractionated immunoblotting further validated the expression of UQCC3 protein in the nucleus under hypoxia. The gradual decrease of oxygen levels from 21% to 1% and prolonged exposure to hypoxia led to an increase in UQCC3 protein levels in mitochondria, and the translocation to nuclei (Data not shown). These findings suggest that UQCC3 is located in the nucleus under hypoxia.

**Fig. 1:**
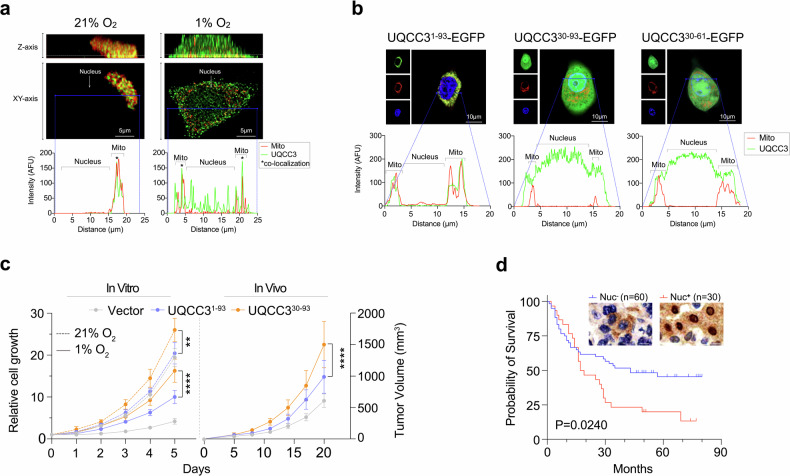
Nuclear localization of UQCC3 driving tumor adaptation to hypoxia predicts adverse clinical outcomes. **a** Nuclear localization of UQCC3 protein. Immunofluorescent imaging of UQCC3-EGFP in HepG2 cells in normoxia and hypoxia. **b** The analysis and function of putative bipartite NLS in UQCC3. Immunofluorescent images and distribution analysis of indicated UQCC3-EGFP fusion proteins in HepG2 cells. **c** The nuclear localization of UQCC3 promotes HCC cell growth. Growth curves comparing various length of UQCC3-overexpressing HepG2 cells cultured under normoxic and hypoxic conditions in vitro (Left), and HepG2 xenograft tumors in vivo (Right). ^**^P < 0.01, ****P < 0.0001. **d** The nuclear localization of UQCC3 associating to poor survival. Kaplan-Meir survival curve analyses, along with representative immunohistochemical staining images, reveal differences in survival rates between HCC patients with positive and negative nuclear staining of UQCC3 protein. Scale bar: 10 μm

To determine the mechanism of UQCC3 translocation into nuclei, we used cNLS mapper to predict nuclear localization signal (NLS) of UQCC3. The NLS, located at amino acids 30–61, was a bipartite NLS, which is characterized by two clusters of 2–3 basic amino acids that are separated by a 9-12 amino acid linker region containing several proline residues.^4^ To validate the functional role of this NLS, we constructed EGFP fusion proteins with the full-length UQCC3 (1–93), the NLS sequence (30–61), and a longer sequence encompassing the NLS (30–93). We found that the full-length UQCC3 fusion protein was primarily localized in the mitochondria, whereas the NLS sequence alone and the longer sequence efficiently directed EGFP fusion proteins to the nucleus under normoxia. Fluorescence intensity analysis showed that UQCC3 was not colocalized in mitochondria, but had higher intensity in the nucleus compared to the cytoplasm (Fig. 1b). These findings confirm the functional role of the putative NLS in UQCC3.

To gain a deeper understanding of how UQCC3 was targeted to the nucleus, we conducted subcellular fractionation experiments along with immunoblotting. The overexpression of UQCC3^1-93^-EGFP remains a fragment (37 kDa; full-length) only in mitochondria despite the oxygen condition, another cleaved fragment (<35 kDa) was found in both mitochondria and nucleus under hypoxia. The UQCC3^30-93^-EGFP fusion protein, exclusively localized within the nucleus, resembled this cleaved product in size (Data not shown). This suggests that hypoxia induces cleavage of UQCC3 in mitochondria, thereby promoting its nuclear targeting.

Mitochondrial inner membrane proteases, notably presenilin associated rhomboid like (PARL), preferentially cleave proteins with a valine at 4 residues forward to the cleavage site.^5^ Remarkably, UQCC3 bears such a valine at position 26 within the inner membrane, suggesting that PARL may cleave UQCC3 at position 29, unveiling its NLS, is highly plausible. Western blot analysis confirmed mitochondrial localization of PARL and its upregulation under hypoxia. Silencing PARL eliminated the mitochondrial cleaved band and nuclear translocation of UQCC3. Alanine scanning mutagenesis at positions 26–29 identified these amino acids as crucial for normal mitochondrial cleavage by PARL. Mutations at these positions abolished normal cleavage or led to abnormal and unstable mitochondrial cleavage (Data not shown). These data suggest that PARL is crucial for nuclear localization of UQCC3 under hypoxia, highlighting the importance of amino acids 26–29 in PARL-mediated mitochondrial cleavage of UQCC3.

To examine the functional implications of UQCC3 nuclear expression in hypoxic tumor cells, we established a stable HepG2 cell line overexpressing an empty vector, full-length UQCC3, or the nuclear-targeted UQCC3^30-93^. Under normoxia and hypoxia, UQCC3^30-93^ cells showed a significant growth advantage in cell growth compared with UQCC3^1-93^ cells. This effect was consistent in vivo, where UQCC3^30-93^-overexpressing xenografts demonstrated a marked growth advantage, reaching a mean tumor volume of 1501.15 mm^3^ by day 20 compared with 987.90 mm^3^ in UQCC3^1-93^-overexpressing xenografts (Fig. 1c). These findings emphasize the proliferative potential of NLS-localized UQCC3 in tumors.

To clarify the clinical relevance of nuclear UQCC3 in HCC, we stained for UQCC3 on a tissue array comprising tumor and adjacent tissues (total of 180 spots) from 90 patients with HCC. We found that 30 (33.3%) tumor samples had positive staining in nuclei. A survival analysis showed a significant decrease in the median survival time in patients with nuclear-positive UQCC3 (18 months) compared with patients with nuclear-negative UQCC3 (43 months) (Fig. 1d, *P* = 0.0240). These results indicate the nuclear translocation of UQCC3 in HCC tumor tissues and suggest that the nuclear localization of UQCC3 is significantly associated with poorer prognosis.

Together, we discovered a novel mitochondrial retrograde signaling and revealed the role of mitochondrial UQCC3 as a novel nuclear factor that affects tumor adaptation to hypoxia, improving our understanding of the intricate interplay between the mitochondria and the nucleus. Our study highlights that nuclear UQCC3 may serve as a prognostic biomarker and therapeutic target for liver cancer.

### Supplementary information

Hypoxia-induced nuclear localization of ubiquinol-cytochrome-c reductase complex assembly factor 3 (UQCC3) in hepatocellular carcinoma

## Data Availability

The data and materials used in the current study are available from the corresponding authors upon reasonable request.
