# Supplementary material for: Hypoxia-induced nuclear localization of ubiquinol-cytochrome-c reductase complex assembly factor 3 (UQCC3) in hepatocellular carcinoma
Source: Signal Transduct Target Ther. 2024 Jul 29;9:191. doi: 10.1038/s41392-024-01909-x (PMC11284201; doi:10.1038/s41392-024-01909-x)
Supplement: Supplementary file 1 — Hypoxia-induced nuclear localization of ubiquinol-cytochrome-c reductase complex assembly factor 3 (UQCC3) in hepatocellular carcinoma [file 41392_2024_1909_MOESM1_ESM.docx]

Supplementary Materials for

**Hypoxia-induced nuclear localization of ubiquinol-cytochrome-c reductase complex assembly factor 3 (UQCC3) in hepatocellular carcinoma**

Yun Yang^1^, Yinhao Wei^1^, Yiying Sun^1^, Yong Zhou^2,3^, Hanshuo Yang^1,3,4^

Correspondence to: nutritioner@hotmail.com (Y.Z.) and yhansh@scu.edu.cn (H.Y.)

**This file includes:**

Materials and Methods

**Materials and Methods**

**Histology**

Human hepatocellular carcinoma (HCC) tissue-Chip was procured from Shanghai Outdo Biotech (Shanghai, China). Human HCC samples, xenografts, and spontaneous liver cancer tissues were fixed in 10% formaldehyde solution (Sigma), processed, embedded in paraffin, and sectioned into 4 μM slices. H&E or IHC staining was conducted at the Laboratory of Pathology, West China Hospital, Sichuan University, China. To ascertain antibody specificity, PBS replaced the primary antibody (Sigma, HPA046851) as a negative control. Images were examined and representative areas exported using CaseViewer (3DHISTECH). Image Pro Plus 6.0 software was employed to determine the density of IHC Images.

**Cell Imaging and co-localization analysis**

HepG2 cells were transfected with the UQCC3-EGFP plasmid using Lipofectamine 3000 (Thermo Fisher). Forty-eight hours post-transfection, the cells were passage into confocal culture dishes (NEST). These dishes were then maintained under both normoxic (21% oxygen) and hypoxic (1% oxygen) conditions for a duration of 24 hours. To facilitate the visualization of mitochondria and the nucleus, MitoTracker Red (100 nM, Invitrogen) was used for 30 minutes, and Hoechst 33342 staining solution (10 μg/mL, Solarbio) was applied for 10 minutes as markers, respectively. Imaging was executed using a Zeiss 980 laser confocal microscope. The microscope was set to capture images in the mid-plane of the cell along the z-axis, specifically focusing on a plane intersecting both mitochondria and the nucleus (Figure 1a, Lower-right). This approach permitted a direct fluorescence intensity comparison between these organelles. A 100X 1.3 oil objective lens, coupled with a 2X zoom, offered a high-resolution view, resulting in a fine pixel size of 0.034 μm x 0.034 μm x 0.110 μm. Initial co-localization analysis was conducted using ZEN imaging software (Zeiss). For a more detailed quantitative assessment, Image J software was employed. TIFF images exported from the ZEN software were processed using “Color” and “Channel tool” module of Image J to segment the three-color channels. A specified line intersecting the mitochondria and nucleus was selected, and fluorescence intensity profiles for EGFP and MitoTracker were measured using the “Plot Profile” setting in Image J. This process enabled us to directly compare the signal intensities of these two fluorophores. Importantly, the absence of MitoTracker Red signal in the nuclear portion of the selected plane served as evidence for the purity of the UQCC3-EGFP nuclear signal. The quantitative data obtained from Image J was then transferred to Graphpad for plotting and further analysis. This methodology facilitated the precise visualization and quantification of UQCC3 localization in the lower-mid plane of the nucleus.

**Plasmid Constructs and Lentivirus Packaging**

The full-length coding sequence (CDS) of the human UQCC3 gene, specifically fragments 1-93 and 30-93, were cloned into the pWPXLD vector (Addgene) utilizing seamless cloning technology (Beyotime, #D7010M). Additionally, an empty pWPXLD vector was prepared to serve as a control. Lentiviruses carrying the cloned full-length or truncated UQCC3 genes (or the empty vector as a control) were generated by co-transfecting 293T packaging cells with the constructed plasmids alongside two helper plasmids: psPAX2 (packaging plasmid) and pMD2.G (envelope plasmid). Following transfection, the supernatant rich in lentiviruses was carefully collected. This supernatant underwent purification to eliminate any potential contaminants, ensuring a high-quality virus preparation for subsequent infection. The purified lentiviruses were subsequently used to infect HepG2 cells. The purified virus was diluted 100 times in fresh media supplemented with polybrene (5 μg/ml) and used to incubate HepG2 Cells, which were subsequently centrifuged for 1 hour at 1000g at 32°C.

**Cell Counting**

HepG2 cells, stably transduced with lentivirus to overexpress 1-93, 30-93 or not (empty vector) confirmed via western blot and qRT-PCR, were maintained in Dulbecco's modified Eagle's medium (Gibco) supplemented with 10% fetal bovine serum (Gibco) and antibiotics. The cells were incubated at 37°C in a humidified atmosphere containing 5% CO_2_. To assess cellular proliferation rates, the HepG2 cells overexpressing 1-93 or 30-93 were seeded at a density of 20,000 cells per well in 24-well plates on Day 0. The plates were then incubated under two different oxygen concentrations: 21% and 1%. Daily, three biologically replicated wells from each group were harvested and digested for cell counting. Cell counts were determined using the Countstar® Bio Tech system, and proliferation was expressed as a relative cell growth or "Fold Change," normalized to the initial seeding cell number on Day 0, which was set as "1." This normalization allowed for a direct comparison of growth rates between different oxygen conditions.

**Xenograft Models**

Xenograft models were established using nude mice (Gempharmatech Co., Ltd. Nanjing, China). Each mouse underwent subcutaneous inoculation with 5 million HepG2 cells that had been stably transfected with lentivirus expressing either an empty vector, 1-93 UQCC3, or 30-93 UQCC3. Western blot analysis and qRT-PCR were performed to validate the overexpression of both lengths of UQCC3 (1-93 and 30-93). The day of tumor inoculation was designated as Day 0. Tumor volume dynamics were closely monitored at three-day intervals, commencing five days post-inoculation. The tumor volumes were calculated using the formula: 0.52 × length × width^2. This method allowed for a consistent and accurate assessment of tumor growth over time. This study included eight mice per group. The experimental animal scheme strictly complied with the requirements of the experimental animal ethics committee of SiChuan University and conformed to the principles of animal protection, animal welfare, and ethics. These principles ensured the humane treatment of the animals throughout the duration of the study.

**Statistical Analysis**

All the experimental data analyses were performed with GraphPad Prism statistical software and shown as mean ± SEM. Two-sample t-tests and two-way analysis of variance were employed for group comparisons. Survival analyses were conducted using Kaplan-Meier curves and the log-rank test. A significance level of P<0.05 was considered statistically significant.
